# Impact of adopting the 2013 World Health Organization criteria for diagnosis of gestational diabetes in a multi-ethnic Asian cohort: a prospective study

**DOI:** 10.1186/s12884-018-1707-3

**Published:** 2018-03-21

**Authors:** Claudia Chi, See Ling Loy, Shiao-Yng Chan, Cherie Choong, Shirong Cai, Shu E. Soh, Kok Hian Tan, Fabian Yap, Peter D. Gluckman, Keith M. Godfrey, Lynette Pei-Chi Shek, Jerry Kok Yen Chan, Michael S. Kramer, Yap-Seng Chong

**Affiliations:** 1 Department of Obstetrics & Gynaecology, National University Hospital, Singapore, 119074 Singapore; 2 Department of Reproductive Medicine, KK Women’s and Children’s Hospital, Singapore, 229899 Singapore; 3 Duke-NUS Medical School, Singapore, 169857 Singapore; 4 Singapore Institute for Clinical Sciences, Agency for Science, Technology and Research (A*STAR), Singapore, 117609 Singapore; 5 Department of Obstetrics & Gynaecology, Yong Loo Lin School of Medicine, National University of Singapore, National University Health System, Singapore, 119228 Singapore; 6 Department of Paediatrics, Yong Loo Lin School of Medicine, National University of Singapore, Singapore, 119228 Singapore; 7 Department of Maternal Fetal Medicine, KK Women’s and Children’s Hospital, Singapore, 229899 Singapore; 8 Department of Paediatrics, KK Women’s and Children’s Hospital, Singapore, 229899 Singapore; 9 Lee Kong Chian School of Medicine, Nanyang Technological University, Singapore, 636921 Singapore; 10 Liggins Institute, University of Auckland, Auckland, 1142 New Zealand; 11 Medical Research Council Lifecourse Epidemiology Unit, University of Southampton, Southampton, SO16 6YD UK; 12 National Institute for Health Research Southampton Biomedical Research Centre, University of Southampton and University Hospital Southampton National Health Service Foundation Trust, Southampton, SO16 6YD UK; 13 Khoo Teck Puat-National University Children’s Medical Institute, National University Hospital, National University Health System, Singapore, 119074 Singapore; 14 Department of Pediatrics, McGill University Faculty of Medicine, 845 Rue Sherbrooke Ouest, Montreal, QC, H3A 0G4 Canada; 15 Department of Epidemiology, Biostatistics and Occupational Health, McGill University Faculty of Medicine, 845 Rue Sherbrooke Ouest, Montreal, QC, H3A 0G4 Canada

**Keywords:** Gestational diabetes mellitus, Glucose tolerance, Pregnancy outcomes, Type 2 diabetes, World Health Organization

## Abstract

**Background:**

We assessed the impact of adopting the 2013 World Health Organization (WHO) diagnostic criteria on the rates of gestational diabetes (GDM), pregnancy outcomes and identification of women at future risk of type 2 diabetes.

**Methods:**

During a period when the 1999 WHO GDM criteria were in effect, pregnant women were universally screened using a one-step 75 g 2-h oral glucose tolerance test at 26–28 weeks’ gestation. Women were retrospectively reclassified according to the 2013 criteria, but without the 1-h glycaemia measurement. Pregnancy outcomes and glucose tolerance at 4–5 years post-delivery were compared for women with GDM classified by the 1999 criteria alone, GDM by the 2013 criteria alone, GDM by both criteria and without GDM by both sets of criteria.

**Results:**

Of 1092 women, 204 (18.7%) and 142 (13.0%) were diagnosed with GDM by the 1999 and 2013 WHO criteria, respectively, with 27 (2.5%) reclassified to GDM and 89 (8.2%) reclassified to non-GDM when shifting from the 1999 to 2013 criteria. Compared to women without GDM by both criteria, cases reclassified to GDM by the 2013 criteria had an increased risk of neonatal jaundice requiring phototherapy (relative risk (RR) = 2.78, 95% confidence interval (CI) 1.32, 5.86); despite receiving treatment for GDM, cases reclassified to non-GDM by the 2013 criteria had higher risks of prematurity (RR = 2.17, 95% CI 1.12, 4.24), neonatal hypoglycaemia (RR = 3.42, 95% CI 1.04, 11.29), jaundice requiring phototherapy (RR = 1.71, 95% CI 1.04, 2.82), and a higher rate of abnormal glucose tolerance at 4–5 years post-delivery (RR = 3.39, 95% CI 2.30, 5.00).

**Conclusions:**

Adoption of the 2013 WHO criteria, without the 1-h glycaemia measurement, reduced the GDM rate. Lowering the fasting glucose threshold identified women who might benefit from treatment, but raising the 2-h threshold may fail to identify women at increased risk of adverse pregnancy and future metabolic outcomes.

**Trial registration:**

NCT01174875. Registered 1 July 2010 (retrospectively registered).

**Electronic supplementary material:**

The online version of this article (10.1186/s12884-018-1707-3) contains supplementary material, which is available to authorized users.

## Background

Gestational diabetes mellitus (GDM), defined as any degree of glucose intolerance with onset or first recognition during pregnancy [1], is associated with adverse outcomes in mothers and offspring [2, 3]. However, international uniformity for the ascertainment and diagnosis of GDM has not been reached and has remained a contentious issue [4]. In 2010, the International Association of Diabetes and Pregnancy Study Group (IADPSG) proposed a new set of diagnostic criteria for GDM [5] based on the graded dose-response associating maternal glycaemia with pregnancy outcomes reported in the Hyperglycaemia and Adverse Pregnancy Outcomes (HAPO) Study [2]. It was recommended that GDM be diagnosed if one or more of the following criteria are met: fasting plasma glucose (FPG) 5.1–6.9 mmol/L, 1-h plasma glucose (PG) ≥ 10.0 mmol/L or 2-h PG 8.5–11.0 mmol/L [5, 6]; whilst those with FPG ≥ 7.0 mmol/L or 2-h PG ≥ 11.1 mmol/L are diagnosed with diabetes in pregnancy [6]. The thresholds for GDM diagnosis were the glucose values at which the odds for birth weight, cord C peptide concentration and percentage of newborns with neonatal body fat greater than the 90th centile reached 1.75 times the odds of these outcomes at the mean glucose values of the entire study cohort of pregnant women who were deemed not to have pre-existing diabetes [5]. In contrast, the earlier 1999 WHO GDM diagnostic criteria were based on the thresholds used to define diabetes mellitus or impaired glucose tolerance outside pregnancy (FPG > 7.0 mmol/L, 2-h PG ≥7.8 mmol/L) [7], whilst the original O’Sullivan and Mahan criteria (FPG ≥5.8 mmol/L, 1-h PG ≥10.6 mmol/L, 2-h PG ≥9.2 mmol/L, 3-h PG ≥8.1 mmol/L) was aimed primarily to identify women at increased risk of developing future type 2 diabetes mellitus [8].

In 2013, the World Health Organization (WHO) endorsed and adopted the IADPSG diagnostic criteria for GDM in an attempt to achieve a universal standard for the diagnosis of GDM [6]. Nevertheless, the use of these new criteria in GDM screening remains controversial as no evidence from prospective randomized studies have shown improved maternal and fetal outcomes with their adoption [9]. In addition, differences in GDM prevalence and in the relative diagnostic importance of FPG, 1-h PG and 2-h PG were observed across the geographically-diverse centres of the HAPO study [10]. Therefore, the WHO has recommended further evaluation of the impact of adopting the 2013 criteria in diverse settings and ethnic groups [6].

In comparison to the 1999 WHO criteria [7], the 2013 criteria used a lower threshold for FPG but a higher threshold for the 2 h PG and the addition of a 1-h PG [6]. A shift from the 1999 WHO criteria to the new 2013 WHO criteria may lead to diagnosis of more cases with increased FPG and/or increased 1-h PG but “miss” cases with slightly increased 2-h PG (i.e. those between 7.8 mmol/L and 8.4 mmol/L). It is therefore important to document the fraction of newly-diagnosed GDM cases and “missed” GDM cases, and to assess their pregnancy outcomes and subsequent metabolic risk. Such information may impact screening strategies and affect the proportion of pregnant women who receive GDM treatment and are followed-up after delivery for future development of type 2 diabetes mellitus.

People of Chinese (74.3%), Malay (13.3%) and Indian (9.1%) ethnicity make up the majority of the population in Singapore [11]. Based on the latest report from the International Diabetes Federation, people of Chinese and Indian ethnicity comprise the world’s largest populations at risk of developing type 2 diabetes mellitus [12]. Because of its ageing and increasingly sedentary population, Singapore presages health problems that other Asian countries are likely to face in the decades ahead [13]. The prevalence of type 2 diabetes mellitus in Singapore has been forecasted to double from 7.3% in 1990 to 15.0% in 2050 [13]. Furthermore, Singapore has one of the highest GDM rates in the world [10].

Women with GDM are at high risk of developing type 2 diabetes mellitus [14]. It is therefore important to study the prevalence of GDM in ethnicities most heavily affected by diabetes. In this study, we used prospectively collected data from a multi-ethnic group of Asian women participating in the Growing Up in Singapore Towards healthy Outcomes (GUSTO) cohort study [15] to compare the prevalence of GDM based on the 1999 and 2013 WHO criteria across three Asian ethnicities, and assess the impact of GDM reclassification switching from the older to the newer criteria on pregnancy outcomes and on glucose tolerance of women 4–5 years after delivery.

## Methods

### Study design and participants

GUSTO is an on-going Asian mother-offspring cohort study [15]. It is conducted according to the guidelines laid down in the Declaration of Helsinki. Ethical approval was obtained from the Centralised Institutional Review Board of SingHealth (reference 2009/280/D) and the Domain Specific Review Board of Singapore National Healthcare Group (reference D/09/021). Informed written consent was obtained from all participants.

Between June 2009 and September 2010, 1247 pregnant women aged 18 and above were recruited during the first trimester of pregnancy (< 14 weeks’ gestation based on a first trimester dating ultrasound scan) from KK Women’s and Children’s Hospital (KKH) and National University Hospital (NUH), the two major public maternity units in Singapore. The recruited women were Singapore citizens or permanent residents from three different ethnic groups (Chinese, Malay, and Asian Indian) with homogenous parental ethnic background. Women receiving chemotherapy or psychotropic drugs or with type 1 diabetes mellitus were excluded. For this study we also excluded women with multiple pregnancies, resulting in 1237 women.

### Data collection

Recruited women returned to the hospitals at 26–28 weeks’ gestation for a follow-up study visit. Detailed interviews and measurements were conducted in the clinics at recruitment and at 26–28 weeks’ gestation. Data on socioeconomic status, family history, obstetric history and smoking status were collected. Family history of diabetes mellitus was based only on first-degree relatives. Smoking status was defined as smoking during the current pregnancy.

Self-reported pre-pregnancy weight and measured booking weight at the first antenatal clinic visits (≤14 weeks’ gestation) were ascertained. Height was measured with a portable stadiometer (Seca 213, Hamburg, Germany) at 26–28 weeks’ gestation. Body mass index (BMI) was determined using the formula of weight (kg)/ height (m^2^). Since early pregnancy BMI at the first clinic visit was free from recall bias, was strongly correlated with pre-pregnancy BMI (*r* = 0.96, *p* < 0.001) and had fewer missing values (0.7%), it was used in all analyses. Gestational weight gain (GWG) was computed as the difference between booking weight and the measured weight at 26–28 weeks’ gestation and expressed as gestational age-specific z-score based on the reference of [16].

Data on pregnancy outcomes were retrieved from the hospital case notes, discharge summaries and electronic medical records by trained health personnel.

### Oral glucose tolerance test

Women underwent a 75-g oral glucose tolerance test (OGTT) after an overnight fast (8 to 10 h) at 26–28 weeks’ gestation and at 4–5 years after delivery. Plasma glucose concentrations at 0 and 120 min following the oral glucose load were measured by colorimetry [Advia 2400 Chemistry system (Siemens Medical Solutions Diagnostics) and Beckman LX20 Pro analyser (Beckman Coulter)]. GDM was diagnosed using the 1999 WHO criteria: ≥7.0 mmol/L for FPG and/ or ≥ 7.8 mmol/L for 2-h PG [7]. Women with GDM were subsequently managed with dietary advice with or without insulin treatment according to standard protocols in both hospitals. Following an OGTT 4–5 years after delivery, type 2 diabetes mellitus (DM), impaired fasting glucose (IFG) and impaired glucose tolerance (IGT) were diagnosed using the 2006 WHO criteria: FPG ≥7.0 mmol/L or 2-h PG ≥11.1 mmol/L for DM; FPG 6.1 to 6.9 mmol/L and 2-h PG < 7.8 mmol/L for IFG; and FPG < 7.0 mmol/L and 2-h PG ≥7.8 and < 11.1 mmol/L for IGT [17].

### GDM diagnostic criteria

GDM was diagnosed using the 1999 WHO criteria (≥7.0 mmol/L for FPG and/ or ≥ 7.8 mmol/L for 2-h PG) [7] during the GUSTO pregnancy period and retrospectively the 2013 WHO criteria (≥5.1 mmol/L for FPG and/ or ≥ 8.5 mmol/L for 2-h PG) [6] was applied for the purposes of this specific study. The 1-h PG included in the 2013 WHO criteria was not measured in our study, as it was not part of the routine OGTT performed at that time. Women were reclassified into four mutually exclusive groups using the 1999 and 2013 WHO GDM diagnostic criteria. Group 1 included women who were classified as non-GDM by both criteria (FPG < 5.1 mmol/L and 2-h PG < 7.8 mmol/L); Group 2 included women who were diagnosed as GDM by the 1999 WHO criteria but reclassified as normal by the 2013 WHO criteria (FPG < 5.1 mmol/L but 2-h PG between ≥7.8 and < 8.5 mmol/L); Group 3 included women who were normal by the 1999 WHO criteria but reclassified as GDM by the 2013 WHO criteria (FPG between ≥5.1 and < 7.0 mmol/L but 2-h PG < 7.8 mmol/L); Group 4 included women who were GDM by both criteria (FPG ≥7.0 mmol/L and/ or 2-h PG ≥8.5 mmol/L).

### Outcome measures

Maternal outcomes included requirement for insulin, induction of labour, caesarean delivery, instrumental vaginal delivery, hypertensive disorders (including chronic hypertension, pregnancy-induced hypertension and pre-eclampsia in the index pregnancy), as well as abnormal glucose tolerance (type 2 DM, IFG and IGT) at 4–5 years after delivery.

Foetal outcomes included gestational age at birth, preterm birth (< 37 completed weeks), Apgar scores at 1 min and 5 min, birth weight-for-gestational-age (birth-weight-for-GA) z-score, small-for-gestational-age (SGA) birth, large-for-gestational-age (LGA) birth, macrosomia (> 4 kg), shoulder dystocia, hypoglycaemia, neonatal jaundice requiring phototherapy and neonatal intensive care unit (NICU) admission. Gestational age was determined based on ultrasound scan measurements at 7–12 weeks. A customized birth weight-for-GA centile chart based on GUSTO data was derived according to Mikolajczyk et al. [18] using solely gestational age for customization. SGA and LGA birth were defined, respectively, as <10th and > 90th centile using the customized birth weight-for-GA centile chart. Hypoglycaemia was defined as a capillary blood glucose ≤2.5 mmol/L.

### Statistical analysis

Statistical analyses were performed using SPSS software, Version 20 (USA) and StataCorp Stata Statistical Software, Release 13 (USA). Categorical data are presented as frequencies and percentages, while continuous data are presented as means and SDs. Comparisons of maternal characteristics and outcomes between groups were performed using Pearson’s Chi-square or Fisher’s exact tests, as appropriate, for categorical variables and independent t-tests for continuous variables. Differences in GDM rates between paired proportions were assessed using McNemar’s test. Modified poisson regression [19] was used to obtain adjusted relative risks (RRs) and 95% confidence intervals (CIs) associated with each GDM classification group for categorical outcomes, while linear regression was used for continuous outcomes with non-GDM women defined as the reference group. Potential confounding variables were identified from the literature [20, 21] and included maternal age, ethnicity, education, body mass index, gestational weight gain, parity, family history of diabetes and type of conception. Additional adjustment for infant sex was performed for LGA and birth weight-for-GA z-score outcomes and adjustment for SGA birth was performed for neonatal hypoglycaemia and jaundice requiring phototherapy.

According to the 2013 WHO criteria, diabetes in pregnancy, as opposed to GDM, is diagnosed when FPG ≥7.0 mmol/L or 2-h PG ≥11.1 mmol/L [6]. In our study, nine women fell into this category. Therefore we also conducted a sensitivity analysis restricting the samples to those with FPG < 7.0 mmol/L and 2-h PG < 11.1 mmol/L (*n* = 1083 for assessing pregnancy outcomes; *n* = 648 for assessing glucose tolerance at 4–5 years post-delivery).

## Results

Of the 1237 women with singleton pregnancies, 1092 (88.3%) underwent OGTT at 26–28 weeks’ gestation. The remaining 145 (11.7%) women either missed their 26–28 weeks study visit, declined testing or had type 2 diabetes mellitus. No significant differences were observed between the 1092 women who had OGTT and the 145 women who did not have OGTT in terms of ethnicity, age, BMI, GWG, education, parity, family history of diabetes, smoking during pregnancy and type of conception.

Table 1 shows the numbers and proportions of women diagnosed with GDM based on the 1999 and 2013 WHO criteria, and reclassification of GDM using the different criteria. Use of the 2013 WHO criteria, (without the 1-h PG) reduced the overall GDM rate from 18.7% (*n* = 204) to 13.0% (*n* = 142) based on universal screening. The greatest reduction in the rate of GDM was observed among Chinese women (from 20.3% to 11.8%; *p* < 0.001), with the same trend among Indians (from 23.8% to 18.5%; *p* = 0.064) but little change among Malay women (from 11.5% to 11.9%; *p* > 0.950). Overall, 89 women with GDM (8.2% of total, and 43.6% of GDM cases based on the 1999 WHO criteria) were reclassified to non-GDM, while 27 (2.5% of total) women without GDM by the 1999 WHO criteria were reclassified to GDM with the 2013 WHO criteria. The diagnosis of GDM remained unchanged in 115 (10.5% of total) women who were diagnosed with GDM by both the 1999 and 2013 WHO criteria.

**Table 1:** Rates of GDM and the reclassification of the diagnosis of GDM using the 1999 and 2013 WHO criteria

	GDM based on 1999 WHO criteria	GDM based on 2013 WHO Criteria^a^	Non-GDM based on both 1999 and 2013 WHO criteria^a^ (Group 1)	Reclassified from GDM to non-GDM using 2013 WHO criteria^a^ (Group 2)	Reclassified from non-GDM to GDM using 2013 WHO criteria (Group 3)	GDM based on both 1999 and 2013 WHO criteria (Group 4)
	*n* (%)	*n* (%)	*n* (%)	*n* (%)	*n* (%)	*n* (%)
Chinese (*n* = 625)	127 (20.3)	74 (11.8)^b^	490 (78.4)	61 (9.8)	8 (1.3)	66 (10.6)
Malay (*n* = 278)	32 (11.5)	33 (11.9)	234 (84.2)	11 (4.0)	12 (4.3)	21 (7.6)
Indian (*n* = 189)	45 (23.8)	35 (18.5)	137 (72.5)	17 (9.0)	7 (3.7)	28 (14.8)
Total (*n* = 1092)	204 (18.7)	142 (13.0)^b^	861 (78.8)	89 (8.2)	27 (2.5)	115 (10.5)

*GDM* gestational diabetes mellitus, *WHO* World Health Organization

^a^without the 1-h plasma glucose measure

^b^differences in GDM rates based on 1999 vs 2013 WHO criteria; *P* < 0.001 based on McNemar’s test

Table 2 compares the characteristics and pregnancy outcomes among the four groups. Baseline characteristics and pregnancy outcomes of women reclassified from GDM to non-GDM (Group 2), from non-GDM to GDM (Group 3) and GDM by both the 1999 and 2013 WHO criteria (Group 4) were compared against women who were non-GDM by both criteria (Group 1). In comparison to Group 1, those in Group 2 were older (32.2 vs 30.2 years, *p* = 0.001) and were more likely to have received higher education (46.1% vs 32.5%, *p* = 0.010). Women in Group 3 had a higher BMI (28.0 vs 23.3 kg/m^2^, *p* < 0.001) and were more likely to be multiparous (74.1% vs 52.9%, *p* = 0.030) and have a positive family history of diabetes (59.3% vs 28.1%, *p* < 0.001). Women in Group 4 were older (32.7 vs 30.2 years, *p* < 0.001), had a higher BMI (25.5 vs 23.3 kg/m^2^, *p* < 0.001), lower GWG z-score (− 1.1 vs − 0.8, *p* = 0.024) and more likely to conceive through in vitro fertilization (14.8% vs 5.9%, *p* < 0.001).

**Table 2:** Characteristics and pregnancy outcomes of women

	Group 1: Non-GDM based on both 1999 and 2013 WHO criteria (*n* = 861)	Group 2: Reclassified from GDM to non-GDM using 2013 WHO criteria (*n* = 89)	Group 3: Reclassified from non-GDM to GDM using 2013 WHO criteria (*n* = 27)	Group 4: GDM based on both 1999 and 2013 WHO criteria (*n* = 115)	*P* value (Group 2 vs. 1)	*P* value (Group 3 vs. 1)	*P* value (Group 4 vs. 1)
Characteristics							
Age, years [mean (SD)]	30.2 (5.1)	32.2 (4.9)	31.2 (5.4)	32.7 (4.6)	0.001	0.348	< 0.001
Body mass index, kgm^−2^ [mean (SD)]	23.3 (4.6)	24.0 (4.5)	28.0 (5.7)	25.5 (4.9)	0.195	< 0.001	< 0.001
Gestational weight gain, z-score [mean (SD)]	−0.8 (1.1)	−0.8 (0.9)	−0.9 (1.2)	−1.1 (1.1)	0.581	0.792	0.024
Education, *n* (%)							
None/ primary/ secondary	573 (67.5)	48 (53.9)	20 (80.0)	70 (60.9)	0.010	0.187	0.157
Tertiary	276 (32.5)	41 (46.1)	5 (20.0)	45 (39.1)			
Multiparous, *n* (%)	450 (52.9)	46 (51.7)	20 (74.1)	70 (60.9)	0.821	0.030	0.109
Positive family history of diabetes, *n* (%)	242 (28.1)	28 (31.5)	16 (59.3)	39 (33.9)	0.504	< 0.001	0.197
Smoking during pregnancy, *n* (%)	23 (2.7)	2 (2.3)	1 (3.8)	1 (0.9)	> 0.950	0.517	0.346
Type of conception, *n* (%)							
Natural	810 (94.1)	83 (93.3)	25 (92.6)	98 (85.2)	0.757	0.673	< 0.001
in vitro fertilization	51 (5.9)	6 (6.7)	2 (7.4)	17 (14.8)			
Maternal Outcomes							
Insulin requirement, *n* (%)	0	5 (5.6)	0	12 (10.4)	< 0.001	–	< 0.001
Induction of labour, *n* (%)	260 (34.9)	36 (50.0)	9 (42.9)	51 (51.5)	0.011	0.448	0.001
Caesarean section, *n* (%)	242 (30.1)	32 (40.0)	11 (40.7)	34 (30.6)	0.069	0.239	0.915
Instrumental delivery, *n* (%)	48 (7.9)	9 (15.8)	0	4 (4.9)	0.049	0.626	0.346
Hypertension and/ or preeclampsia, *n* (%)	57 (6.6)	5 (5.6)	3 (11.1)	16 (13.9)	0.716	0.420	0.005
Neonatal Outcomes							
Gestation at birth, weeks [mean (SD)]	38.4 (1.4)	38.2 (1.4)	38.1 (1.3)	37.9 (2.2)	0.078	0.200	0.008
Prematurity, *n* (%)	52 (6.1)	11 (12.4)	2 (7.4)	13 (11.3)	0.025	0.680	0.038
Apgar score at 1 min [mean (SD)]	8.9 (0.6)	8.8 (0.6)	8.6 (1.6)	8.8 (0.8)	0.297	0.379	0.179
Apgar score at 5 min [mean (SD)]	9.0 (0.1)	9.0 (0.1)	8.9 (0.6)	9.0 (0.3)	0.456	0.369	0.569
Birth weight-for-gestational age, z-score [mean (SD)]	0.1 (1.1)	0.1 (1.1)	0.7 (1.2)	0.5 (1.6)	0.861	0.006	0.012
Small for gestational age, *n* (%)	91 (10.7)	10 (11.2)	1 (3.7)	11 (9.6)	0.881	0.348	0.706
Large for gestational age, *n* (%)	124 (14.6)	14 (15.7)	6 (22.2)	25 (21.7)	0.776	0.271	0.047
Macrosomia, *n* (%)	15 (1.8)	1 (1.1)	1 (3.7)	2 (1.7)	> 0.950	0.397	> 0.950
Shoulder dystocia, *n* (%)	1 (0.1)	2 (2.4)	0	0	0.025	> 0.950	> 0.950
Hypoglycaemia, *n* (%)	15 (1.9)	6 (7.3)	1 (3.8)	18 (16.2)	0.011	0.413	< 0.001
Neonatal jaundice requiring phototherapy, *n* (%)	86 (11.2)	17 (20.2)	7 (26.9)	22 (20.2)	0.015	0.024	0.007
Neonatal intensive care unit admission, *n* (%)	30 (3.6)	5 (5.7)	2 (7.7)	6 (5.2)	0.368	0.247	0.427

*GDM* gestational diabetes mellitus, *WHO* World Health Organization, *P* value based on independent t-test, Pearson’s Chi-square or Fisher’s exact test, as appropriate

Compared to Group 1, a higher proportion of cases in Group 2 had labour induction (50.0% vs 34.9%, *p* = 0.011), instrumental vaginal delivery (15.8% vs 7.9%, *p* = 0.049), prematurity (12.4% vs. 6.1%, *p* = 0.025), shoulder dystocia (2.4% vs 0.1%, *p* = 0.025), neonatal hypoglycaemia (7.3% vs 1.9%, *p* = 0.011) and neonatal jaundice requiring phototherapy (20.2% vs 11.2%, *p* = 0.015). Newborns of women in Group 3 had a higher mean birth weight-for-GA z-score (0.7 vs 0.1, *p* = 0.006) and were more likely to have jaundice requiring phototherapy (26.9% vs 11.2%, *p* = 0.024) compared to those born to women in Group 1. Treatment with insulin was given to 5.6 and 10.4% of women in Groups 2 and 4, respectively. A higher proportion of cases in Group 4 had labour induction (51.5% vs 34.9%, *p* = 0.001), hypertensive disorders (13.9% vs 6.6%, *p* = 0.005), prematurity (11.3% vs 6.1%, *p* = 0.038), neonatal hypoglycaemia (16.2% vs 1.9%, *p* < 0.001) and neonatal jaundice requiring phototherapy (20.2% vs 11.2%, *p* = 0.007) compared to women in Group 1. Women in Group 4 delivered at an earlier mean gestation (37.9 vs 38.4 weeks, *p* = 0.008) and their newborns had a higher mean birth weight-for-GA z-score (0.5 vs 0.1, *p* = 0.012) compared to women in Group 1. There were no difference in the proportions of SGA births in Groups 2, 3 and 4 compared to Group 1 (Table 2).

Table 3 shows the RRs for associations between GDM reclassification groups and pregnancy outcomes after adjustment for confounding variables. The risks of prematurity, neonatal hypoglycaemia and jaundice requiring phototherapy were 2.17 times (95% CI 1.12, 4.24), 3.42 times (95% CI 1.04, 11.29) and 1.71 times (95% CI 1.04, 2.82) higher, respectively, among women in Group 2 than among those in Group 1, respectively. An increased risk of neonatal jaundice requiring phototherapy [RR = 2.78 (95% CI 1.32, 5.86)] was also observed among newborns of women in Group 3 compared to those in Group 1. Cases in Group 4 had increased risks of prematurity [RR = 2.06 (95% CI 1.10, 3.84)], LGA birth [RR = 1.60 (95% CI 1.07, 2.39)] and neonatal hypoglycaemia [RR = 11.23 (95% CI 4.98, 25.32)]. An increased birth weight-for-GA z-score was observed in women in Group 4 [0.35 (95% CI 0.12, 0.59)] compared with those in Group 1, but not in Groups 2 or 3.

**Table 3:** Associations between reclassification of gestational diabetes mellitus diagnosis and pregnancy outcomes

Pregnancy outcomes	Group 1	Group 2		Group 3		Group 4	
		RR (95% CI)	*P*	RR (95% CI)	*P*	RR (95% CI)	*P*
Hypertension/ preeclampsia^a^	reference	0.80 (0.35, 1.84)	0.596	1.19 (0.46, 3.09)	0.724	1.54 (0.88, 2.70)	0.126
Prematurity^a^	reference	2.17 (1.12, 4.24)	0.023	0.70 (0.10, 4.87)	0.716	2.06 (1.10, 3.84)	0.023
Large for gestational age^b^	reference	1.23 (0.75, 2.03)	0.411	1.14 (0.62, 2.12)	0.667	1.60 (1.07, 2.39)	0.021
Hypoglycaemia^c^	reference	3.42 (1.04, 11.29)	0.044	2.40 (0.31, 18.63)	0.402	11.23 (4.98, 25.32)	< 0.001
Neonatal jaundice requiring phototherapy^c^	reference	1.71 (1.04, 2.82)	0.034	2.78 (1.32, 5.86)	0.007	1.61 (0.98, 2.66)	0.060

RR = relative risk; CI = confidence interval

^a^ Model 1: adjusted for maternal age, ethnicity, education, body mass index, gestational weight gain, parity and family history of diabetes, type of conception

^b^ Model 2: adjusted for same variables as in model 1 + neonatal sex

^c^ Model 3: adjusted for same variables as in model 1 + small-for-gestational-age birth

Table 4 shows the prevalence of abnormal glucose tolerance in the subset of women who consented to an OGTT at 4–5 years after delivery. Women who underwent OGTT at 4–5 years after delivery were older (*p* < 0.001) and more likely to be multiparous (*p* = 0.025) than those who did not; these were adjusted for in subsequent analyses. Of the 653 women who underwent OGTT, 124 (19.0%) had abnormal glucose tolerance, with higher proportions observed among women in Groups 2 (42.6%) and 4 (45.2%) (*p* < 0.001) (Table 4). Women in Groups 2 and 4 had three-fold increased adjusted risks of developing abnormal glucose tolerance by 4–5 years after delivery compared to women in Group 1 [Group 2: RR = 3.39 (95% CI 2.30, 5.00); Group 4: RR = 2.87 (95% CI 1.93, 4.27)]. However, the trend of an increased risk for women in Group 3 was not statistically significant, perhaps due to the small number [Group 3: RR 1.90 (95% CI 0.88, 4.11)].

**Table 4:** Abnormal glucose tolerance development of women by 4 to 5 years after delivery (*n* = 653)

Diabetes status	Group 1: Non-GDM based on both 1999 and 2013 WHO criteria (*n* = 498)	Group 2: Reclassified from GDM to non-GDM using 2013 WHO criteria (*n* = 61)	Group 3: Reclassified from non-GDM to GDM using 2013 WHO criteria (*n* = 21)	Group 4: GDM based on both 1999 and 2013 WHO criteria (*n* = 73)	*P* value
Normal, *n* (%)	439 (67.2)	35 (5.4)	15 (2.3)	40 (6.1)	< 0.001
Abnormal^a^ *n* (%)	59 (9.0)	26 (4.0)	6 (0.9)	33 (5.1)	

*GDM* gestational diabetes mellitus, *WHO* World Health Organization

^a^Abnormal status included diabetes mellitus (FG ≥7.0 mmol/l or PG ≥11.1 mmol/l), impaired fasting glucose (FG 6.1 to 6.9 mmol/l and PG < 7.8 mmol/l) and impaired glucose tolerance (FG < 7.0 mmol/l and PG ≥7.8 and < 11.1 mmol/l). Abnormal, *n* = 124 (19.0%)

In the sensitivity analyses, with the exclusion of women classified with diabetes in pregnancy by the 2013 WHO criteria (*n* = 9), the results remained similar with minimal changes in the effect estimates for the relationship between GDM reclassification groups and risks of pregnancy outcomes (See, Additional file 1: Table S1; See, Additional file 2: Table S2) and abnormal glucose development postnatally (See, Additional file 3: Table S3).

## Discussion

In the multi-ethnic Asian GUSTO cohort, we found that the adoption of the 2013 WHO criteria, without the 1-h PG, reduced the GDM rate from 18.7 to 13.0%, but with marked ethnic variation. The reduction was specifically observed in Chinese and Indian women but remained unchanged among Malay women. This reduction would lead to “missing” almost half (44%) of the cases which may benefit from GDM intervention during pregnancy to reduce maternal and neonatal morbidity, and from postpartum surveillance for primary prevention and early diagnosis of type 2 diabetes mellitus.

The adoption of the 2013 WHO GDM diagnostic criteria substantially increased the prevalence of GDM in the Caucasian population [22, 23] compared to the 1999 WHO GDM diagnostic criteria. However, reported changes in GDM rates have varied across studies of Asian populations. The 2013 WHO criteria diagnosed more GDM cases in the studies by Pan et al. [21] from China (7.7% vs. 6.8%, *n* = 17,808) and Gilder et al. [24] from Thailand (10.1% vs. 6.6%, *n* = 228), a similar proportion of GDM cases in the study by Sagili et al. [25] from India (12.6% vs. 12.4%, *n* = 1231), and fewer GDM cases in studies by Tran et al. [26] from Vietnam (20.4% vs. 24.3%, *n* = 2772) and Yew et al. [20] from Singapore (21.1% vs. 28.8%, *n* = 855). These disparities and data from our study and others’ [27] support the notion of variation in glycaemic responses to a glucose load in pregnancy among different Asian ethnicities. Pregnant women from East Asia and South Asia have been found to be more insulin-resistant, with poorer homeostatic model assessment (HOMA) β-cell function, than Western Europeans [28], leading to different rates of GDM. Other contributory factors to the variations in the reported change in GDM rates include differences in the study setting (community vs. tertiary-care hospital) and type of screening approach (universal vs. risk-based and one-step vs. two-step screening).

The lower overall GDM rate we observed with a shift from the 1999 to the 2013 WHO criteria mainly resulted from the marked reduction (20.3 to 11.8%) in GDM rate among Chinese women. Only a small proportion (1.3%) of Chinese women had a FPG between 5.1 and 6.9 mmol/L which moved them into the GDM category, while a significantly larger proportion (9.8%) had a 2-h PG between 7.8 and 8.4 mmol/L, moving them out of the GDM category. Similarly, amongst the 15 HAPO centres internationally, Hong Kong Chinese participants had one of the lowest proportion (26%; range 24–74%) of GDM diagnosed by FPG but the highest proportion (29%; range 6–29%) of GDM diagnosed by the 2-h PG [10]. We also observed a similar pattern among women of Indian ethnicity. This is consistent with the findings of several studies on Indian women showing the majority of GDM were detected not by the FPG but by the 2-h PG threshold of ≥7.8 mmol/L based on the 1999 WHO Criteria [25, 29, 30]. Hence the 2-h PG is important in Chinese and Indian women and raising the 2-h PG fails to detect many cases that may benefit from identification and treatment to reduce adverse outcomes.

The benefits of screening for and treating GDM have been demonstrated by two randomized controlled trials. The Australian Carbohydrate Intolerance Study in Pregnant Women (ACHOIS) trial found treatment of GDM women as defined by the 1999 WHO criteria reduced the risk (RR of 0.33, 95% CI 0.14–0.75) of serious perinatal complications (defined as shoulder dystocia, bone fracture, nerve palsy and death) [31]. The second trial from the Maternal Fetal Medicine Units (MFMU) network in the US reported that treatment of mild GDM (FPG < 5.3 mmol/L) reduced mean birth weight and the number of LGA infants [32]. In our study, women who fulfilled the 1999 WHO criteria for GDM received treatment. We are therefore unable to assess the true impact on pregnancy outcomes solely due to change in the 2-h PG threshold. However, treatment of GDM in Group 2 would almost certainly have underestimated the difference in the observed outcomes compared to Group 1. The diagnosis of GDM in Groups 2 and 4 was associated with higher rates of labour induction, as this was part of the standard clinical management of GDM, but it did not result in a significantly higher rate of caesarean delivery compared with the non-GDM cases (Group 1). Women with a 2-h PG in the range of 7.8–8.4 mmol/L (Group 2) would be regarded as normal using the 2013 WHO criteria 2-h PG threshold. We found that five (5.6%) women in Group 2 received insulin treatment, probably reflecting a degree of hyperglycaemia during pregnancy that did not respond to dietary intervention alone; eleven (12.4%) and two (2.4%) offspring of mothers in this group experienced preterm birth and shoulder dystocia at delivery, respectively. Furthermore, the offspring of mothers in this group were at 3.4-fold increased risk of neonatal hypoglycaemia and 1.7-fold increased risk of neonatal jaundice requiring phototherapy compared to those whose mothers did not have GDM by both sets of criteria, even after adjusting for demographic and clinical covariates. Knowledge of the diagnosis of GDM might have created a bias towards the diagnosis of neonatal hypoglycaemia and neonatal jaundice through increased surveillance. However, these complications are likely to be in part related to relative foetal hyperglycaemia in utero, as there was no difference in the proportion of SGA births, a known cofounder in the development of these complications, in Group 2 compared to Group 1.

The FPG threshold of 7.0 mmol/L in the 1999 WHO GDM criteria is regarded as being too high as it is the same as the diagnostic cut-off for type 2 diabetes mellitus in non-pregnant adults [6]. In our study women with FPG between 5.1 and 6.9 mmol/L (Group 3) would be reclassified from non-GDM to GDM under the 2013 criteria. In the absence of treatment, their offspring were at 2.8-fold increased risk of developing jaundice requiring phototherapy compared to non-GDM women by both criteria (Group 1). Although higher percentages of women in Group 3 had hypertensive disorders in pregnancy and LGA infants, the differences were not statistically significant compared with Group 1, perhaps reflecting the small number of women in this group. Nonetheless our results suggest that lowering the FPG threshold might be important in reducing hyperglycaemia-related adverse maternal and neonatal outcomes.

The main strength of our study is our ability to assess the impact of GDM reclassification on identifying women at future risk of type 2 diabetes mellitus. To the best of our knowledge, our study is the first to compare later maternal metabolic outcome based on different GDM diagnostic criteria. In a previous meta-analysis, women who have had GDM using a variety of older diagnostic criteria have been shown to have at least a seven-fold increased risk of developing later type 2 diabetes compared with those who had a normoglycaemic pregnancy [14]. In our study we observed that women with GDM by both criteria (Group 4) were at three-fold increased risk of developing abnormal glucose tolerance within a relatively short interval of 4–5 years after delivery compared to women with no GDM by both criteria. More importantly, women who would not have been identified as GDM by the 2013 criteria but only by the 1999 criteria were also at significantly increased risk (similarly high at three-fold) of abnormal glucose tolerance. The adoption of the 2013 WHO criteria would have failed to identify these women who could benefit from timely intervention to reduce their risk of developing later type 2 diabetes mellitus. Other strengths of our study include its multi-ethnic Asian sample and prospective cohort study design.

Our study also has several limitations. The lack of a 1-h PG value could potentially underestimate the prevalence of GDM using the 2013 criteria by not identifying women with an isolated abnormal 1-h PG. In the HAPO survey, 39% of Singaporean women were diagnosed with GDM based on 1-h PG of ≥10.0 mmol/L [10], whilst a study on South Indians found 14% had elevated 1-h PG value [29]. It is currently not known whether women identified as GDM through an isolated raised 1-h PG value in the 2013 WHO criteria would have had different pregnancy outcomes from those identified using the FPG or 2-h PG thresholds. Moreover, women with an isolated raised 1-h PG would come from Group 1; hence the removal of these cases from the control group could conceivably result in further exaggeration of the difference in pregnancy and metabolic outcomes compared with Groups 2, 3 and 4. However, in the absence of a randomised prospective trial of universal screening comparing the utility of the 1999 with the 2013 WHO criteria, the comparison made in our study provides the best estimates of the consequences of using these two different sets of criteria.

The 2013 WHO criteria represent an important initiative to achieve internationally accepted criteria for the diagnosis of GDM [6]. However, the challenge is that any dichotomous classification of normal vs abnormal glucose tolerance in pregnancy fails to account for the fact that maternal glycaemia shows a continuous, monotonic association with adverse pregnancy outcomes [2]. Furthermore, clear differences in the patterns of insulin resistance, glucose tolerance, and rates of GDM among different ethnic groups have been demonstrated in our and other studies [10, 27].

In 2015, the UK National Institute of Health and Care Excellence (NICE) adopted a different set of GDM diagnostic criteria after considering its own health economic evidence and recommended an intermediate FPG threshold of 5.6 mmol/L (between both WHO criteria) but kept the 2-h PG threshold at ≥7.8 mmol/L as in the 1999 criteria [33]. However, power limitations prevent us from assessing the impact of adopting the NICE recommendation in our cohort as it would have reclassified only seven women from non-GDM to GDM.

## Conclusion

In summary, a shift from the 1999 WHO criteria to the 2013 WHO criteria without a 1-h PG measurement, reduced the rate of GDM in this multi-ethnic Asian cohort. Lowering the fasting threshold as per the new WHO criteria identified women who might benefit from GDM treatment, but raising the 2-h threshold, without the 1-h value, would “miss” women at increased risk of adverse pregnancy and metabolic outcomes. In the absence of the 1-h PG measurements, our data support lowering the FPG but not changing the 2-h PG threshold for our population. Further studies are needed to guide the development of effective strategies for screening and identification of women at risk of adverse pregnancy outcomes and long-term metabolic complications particularly in countries experiencing an epidemic of diabetes, like Singapore.

## Additional files

Additional file 1: **Table S1.** Modified poisson regression models of the associations between reclassification of gestational diabetes mellitus diagnosis and pregnancy outcomes, with the inclusion of women without diabetes in pregnancy. (DOCX 13 kb)

Additional file 2: **Table S2.** Linear regression models of the associations between reclassification of gestational diabetes mellitus diagnosis and birth weight-for-GA, with the inclusion of women without diabetes in pregnancy. (DOCX 12 kb)

Additional file 3: **Table S3.** Abnormal glucose tolerance development of women by 4 to 5 years after delivery, with the inclusion of women without diabetes in pregnancy. (DOCX 12 kb)

## Abbreviations

ACHOIS: Australian carbohydrate intolerance study in pregnant women
BMI: Body mass index
CI: Confidence interval
DM: Diabete mellitus
FG: Fasting glucose
FPG: Fasting plasma glucose
GA: Gestational age
GDM: Gestational diabetes mellitus
GUSTO: Growing up in singapore towards healthy outcomes
GWG: Gestational weight gain
HAPO: Hyperglycaemia and adverse pregnancy outcome
HOMA: Homeostatic model assessment
IADPSG: International association of diabetes and pregnancy study group
IFG: Impaired fasting glucose
IGT: Impaired glucose tolerance
LGA: Large-for-gestational-age
MFMU: Maternal fetal medicine unit
NICE: National institute of health care excellence
NICU: Neonatal intensive care unit
OGTT: Oral glucose tolerance test
PG: Plasma glucose
RR: Relative risk
SD: Standard deviation
SGA: Small-for-gestational-age
WHO: World Health Organisation

## References

[1] American Diabetes Association (2014). Diagnosis and classification of diabetes. Diabetes Care.

[2] Metzger BE, Lowe LP, Dyer AR, Trimble ER, Chaovarindr U, Coustan DR (2008). Hyperglycemia and adverse pregnancy outcomes. N Engl J Med.

[3] Retnakaran R, Qi Y, Sermer M, Connelly PW, Hanley AJ, Zinman B (2008). Glucose intolerance in pregnancy and future risk of pre-diabetes or diabetes. Diabetes Care.

[4] Weinert LS (2010). International Association of Diabetes and Pregnancy Study Groups recommendations on the diagnosis and classification of hyperglycemia in pregnancy: comment to the International Association of Diabetes and Pregnancy Study Groups Consensus Panel. Diabetes Care.

[5] Metzger BE, Gabbe SG, Persson B, Buchanan TA, Catalano PA, International Association of Diabetes Pregnancy Study Groups Consensus Panel (2010). International Association of Diabetes and Pregnancy Study Groups recommendations on the diagnosis and classification of hyperglycemia in pregnancy. Diabetes Care.

[6] World Health Organization (2014). Diagnostic criteria and classification of hyperglycaemia first detected in pregnancy: a World Health Organization guideline. Diabetes Res Clin Pract.

[7] Alberti KG, Zimmet PZ (1998). Definition, diagnosis and classification of diabetes mellitus and its complications. Part 1: diagnosis and classification of diabetes mellitus provisional report of a WHO consultation. Diabet Med.

[8] O’sullivan JB, Mahan CM (1964). Criteria for oral glucose tolerance test in pregnancy. Diabetes.

[9] Cundy T, Ackermann E, Ryan EA (2014). Gestational diabetes: new criteria may triple the prevalence but effect on outcomes is unclear. BMJ.

[10] Sacks DA, Hadden DR, Maresh M, Deerochanawong C, Dyer AR, Metzger BE (2012). Frequency of gestational diabetes mellitus at collaborating centers based on IADPSG consensus panel-recommended criteria: the hyperglycemia and adverse pregnancy outcome (HAPO) study. Diabetes Care.

[11] Department of Statistics Singapore. Population Trends 2015. Department of Statistics, Ministry of Trade & Industry, Republic of Singapore. http://www.singstat.gov.sg/publications/publications-and-papers/population-and-population-structure/population-trends. Accessed 23 Feb 2016.

[12] International Diabetes Federation. Diabetes Atlas. 7th. Brussels: International Diabetes Federation. 2015. http://www.diabetesatlas.org/. Accessed 17 Feb 2016.

[13] Phan TP, Alkema L, Tai ES, Tan KH, Yang Q, Lim WY (2014). Forecasting the burden of type 2 diabetes in Singapore using a demographic epidemiological model of Singapore. BMJ Open Diabetes Res Care.

[14] Bellamy L, Casas JP, Hingorani AD, Williams D (2009). Type 2 diabetes mellitus after gestational diabetes: a systematic review and meta-analysis. Lancet.

[15] Soh SE, Tint MT, Gluckman PD, Godfrey KM, Rifkin-Graboi A, Chan YH (2014). Cohort profile: growing up in Singapore towards healthy outcomes (GUSTO) birth cohort study. Int J Epidemiol.

[16] Hutcheon JA, Platt RW, Abrams B, Himes KP, Simhan HN, Bodnar LM (2013). A weight-gain-for-gestational-age z score chart for the assessment of maternal weight gain in pregnancy. Am J Clin Nutr.

[17] World Health Organization (2006). Definition and diagnosis of diabetes mellitus and intermediate hyperglycemia.

[18] Mikolajczyk RT, Zhang J, Betran AP, Souza JP, Mori R, Gulmezoglu AM (2011). A global reference for fetal-weight and birthweight percentiles. Lancet.

[19] Zou G (2004). A modified poisson regression approach to prospective studies with binary data. Am J Epidemiol.

[20] Yew TW, Khoo CM, Thai AC, Kale AS, Yong EL, Tai ES (2014). The prevalence of gestational diabetes mellitus among Asian females is lower using the new 2013 World Health Organization diagnostic criteria. Endocr Pract.

[21] Pan L, Leng J, Liu G, Zhang C, Liu H, Li M (2015). Pregnancy outcomes of Chinese women with gestational diabetes mellitus defined by the IADPSG's but not by the 1999 WHO’s criteria. Clin Endocrinol.

[22] Moses RG, Morris GJ, Petocz P, San Gil F, Garg D (2011). The impact of potential new diagnostic criteria on the prevalence of gestational diabetes mellitus in Australia. Med J Aust.

[23] O’Sullivan EP, Avalos G, O’Reilly M (2011). Atlantic diabetes in pregnancy (DIP): the prevalence and outcomes of gestational diabetes mellitus using the new diagnostic criteria. Diabetologia.

[24] Gilder ME, Zin TW, Wai NS, Ner M, Say PS, Htoo M (2014). Gestational diabetes mellitus prevalence in Maela refugee camp on the Thai-Myanmar border: a clinical report. Glob Health Action.

[25] Sagili H, Kamalanathan S, Sahoo J, Lakshminarayanan S, Rani R, Jayalakshmi D (2015). Comparison of different criteria for diagnosis of gestational diabetes mellitus. Indian J Endocrinol Metab.

[26] Tran TS, Hirst JE, Do MA, Morris JM, Jeffery HE (2013). Early prediction of gestational diabetes mellitus in Vietnam: clinical impact of currently recommended diagnostic criteria. Diabetes Care.

[27] Chu SY, Abe K, Hall LR, Kim SY, Njoroge T, Qin C (2009). Gestational diabetes mellitus: all Asians are not alike. Prev Med.

[28] Morkrid K, Jenum AK, Sletner L, Vardal MH, Waage CW, Nakstad B (2012). Failure to increase insulin secretory capacity during pregnancy-induced insulin resistance is associated with ethnicity and gestational diabetes. Eur J Endocrinol.

[29] Nallaperumal S, Bhavadharini B, Mahalakshmi MM, Maheswari K, Jalaja R, Moses A (2013). Comparison of the World Health Organization and the International Association of Diabetes and Pregnancy Study Groups criteria in diagnosing gestational diabetes mellitus in south Indians. Indian J Endocrinol Metab..

[30] Seshiah V, Balaji V, Shah SN, Joshi S, Das AK, Sahay BK (2012). Diagnosis of gestational diabetes mellitus in the community. J Assoc Physicians India.

[31] Crowther CA, Hiller JE, Moss JR, McPhee AJ, Jeffries WS, Robinson JS (2005). Effect of treatment of gestational diabetes mellitus on pregnancy outcomes. N Engl J Med.

[32] Landon MB, Spong CY, Thom E, Carpenter MW, Ramin SM, Casey B (2009). A multicenter, randomized trial of treatment for mild gestational diabetes. N Engl J Med.

[33] National institute for Health and Care Excellence. Diabetes in pregnancy: management from preconception to the postnatal period. 2015. https://www.nice.org.uk/guidance/ng3. Accessed 17 Feb 2016. 25950069

