# Supplementary material for: Impact of adopting the 2013 World Health Organization criteria for diagnosis of gestational diabetes in a multi-ethnic Asian cohort: a prospective study
Source: BMC Pregnancy Childbirth. 2018 Mar 21;18:69. doi: 10.1186/s12884-018-1707-3 (PMC5863481; doi:10.1186/s12884-018-1707-3)
Supplement: Supplementary file 1 — Table S1. Modified poisson regression models of the associations between reclassification of gestational diabetes mellitus diagnosis and pregnancy outcomes, with the inclusion of women without diabetes in pregnancy. (DOCX 13 kb) [file 12884_2018_1707_MOESM1_ESM.docx]

**Supplementary Table 1** Modified poisson regression models of the associations between reclassification of gestational diabetes mellitus diagnosis and pregnancy outcomes, with the inclusion of women without diabetes in pregnancy

| Pregnancy outcomes | Group 1 | Group 2 |  | Group 3 |  | Group 4 |  |
| --- | --- | --- | --- | --- | --- | --- | --- |
|  |  | RR (95% CI) | P | RR (95% CI) | P | RR (95% CI) | P |
| Hypertension/ preeclampsia^a^ | reference | 0.80 (0.35, 1.83) | 0.595 | 1.16 (0.45, 3.01) | 0.755 | 1.55 (0.86, 2.79) | 0.145 |
| Prematurity^a^ | reference | 2.19 (1.12, 4.27) | 0.022 | 0.71 (0.10, 5.01) | 0.734 | 2.16 (1.12, 4.17) | 0.021 |
| Large for gestational age^b^ | reference | 1.24 (0.75, 2.04) | 0.401 | 1.14 (0.62, 2.11) | 0.676 | 1.46 (0.95, 2.26) | 0.088 |
| Hypoglycaemia^c^ | reference | 3.50 (1.05, 11.68) | 0.042 | 2.32 (0.30, 17.92) | 0.418 | 10.84 (4.51, 26.05) | <0.001 |
| Neonatal jaundice requiring phototherapy^c^ | reference | 1.69 (1.03, 2.79) | 0.038 | 2.70 (1.29, 5.69) | 0.009 | 1.37 (0.79, 2.39) | 0.266 |

RR = relative risk; CI = confidence interval

^a^Model 1: adjusted for maternal age, ethnicity, education, body mass index, gestational weight gain, parity and family history of diabetes, type of conception

^b^Model 2: adjusted for same variables as in model 1 + neonatal sex

^c^Model 3: adjusted for same variables as in model 1 + small-for-gestational-age birth
