# Supplementary material for: Impact of adopting the 2013 World Health Organization criteria for diagnosis of gestational diabetes in a multi-ethnic Asian cohort: a prospective study
Source: BMC Pregnancy Childbirth. 2018 Mar 21;18:69. doi: 10.1186/s12884-018-1707-3 (PMC5863481; doi:10.1186/s12884-018-1707-3)
Supplement: Supplementary file 2 — Table S2. Linear regression models of the associations between reclassification of gestational diabetes mellitus diagnosis and birth weight-for-GA, with the inclusion of women without diabetes in pregnancy. (DOCX 12 kb) [file 12884_2018_1707_MOESM2_ESM.docx]

**Supplementary Table 2** Linear regression models of the associations between reclassification of gestational diabetes mellitus diagnosis and birth weight-for-GA, with the inclusion of women without diabetes in pregnancy^a^

| Pregnancy outcomes | Group 1 | Group 2 |  | Group 3 |  | Group 4 |  |
| --- | --- | --- | --- | --- | --- | --- | --- |
|  |  | β (95% CI) | p | β (95% CI) | p | β (95% CI) | p |
| Birth weight-for-GA (z-score) | reference | -0.09 (-0.36, 0.17) | 0.478 | 0.35 (-0.12, 0.82) | 0.142 | 0.32 (0.08, 0.57) | 0.009 |

GA = gestational age; β = beta coefficient; CI = confidence interval

^a^Adjusted for maternal age, ethnicity, education, body mass index, gestational weight gain, parity, family history of diabetes, type of conception, neonatal sex
