# Supplementary material for: Impact of adopting the 2013 World Health Organization criteria for diagnosis of gestational diabetes in a multi-ethnic Asian cohort: a prospective study
Source: BMC Pregnancy Childbirth. 2018 Mar 21;18:69. doi: 10.1186/s12884-018-1707-3 (PMC5863481; doi:10.1186/s12884-018-1707-3)
Supplement: Supplementary file 3 — Table S3. Abnormal glucose tolerance development of women by 4 to 5 years after delivery, with the inclusion of women without diabetes in pregnancy. (DOCX 12 kb) [file 12884_2018_1707_MOESM3_ESM.docx]

**Supplementary Table 3** Abnormal glucose tolerance development of women by 4 to 5 years after delivery, with the inclusion of women without diabetes in pregnancy^a^

| Diabetes status | Group 1 | Group 2 |  | Group 3 |  | Group 4 |  |
| --- | --- | --- | --- | --- | --- | --- | --- |
|  |  | RR (95% CI) | p | RR (95% CI) | p | RR (95% CI) | p |
| Abnormal^b^ | reference | 3.38 (2.29, 4.99) | <0.001 | 1.92 (0.89, 4.16) | 0.097 | 2.77 (1.82, 4.20) | <0.001 |

RR = relative risk; CI = confidence interval

^a^Adjusted for maternal age, ethnicity, education, body mass index, gestational weight gain, parity, family history of diabetes and type of conception.

^b^Abnormal status included diabetes mellitus (FG ≥7.0mmol/l or PG ≥11.1mmol/l), impaired fasting glucose (FG 6.1 to 6.9mmol/l and PG <7.8mmol/l) and impaired glucose tolerance (FG <7.0mmol/l and PG ≥7.8 and <11.1mmol/l)
